# Exploring Vocational Training Interventions for Adults With Autism Spectrum Disorders: A Scoping Review

**DOI:** 10.1155/oti/7704446

**Published:** 2026-04-06

**Authors:** Haura Alia Herlitasari, Dini Fajariani, Reiko Miyamoto

**Affiliations:** ^1^ Department of Occupational Therapy, Graduate School of Human Health Sciences, Tokyo Metropolitan University, Hachioji, Japan, tmu.ac.jp; ^2^ Department of Occupational Therapy, Vocational Education Program, Universitas Indonesia, Depok, Indonesia, ui.ac.id

**Keywords:** autism spectrum disorder, occupational therapy, scoping review, virtual reality interventions, vocational training

## Abstract

Adults with autism spectrum disorder (ASD) continue to face substantial barriers to obtaining and sustaining employment, including challenges related to social communication, understanding workplace expectations, and navigating job interviews, which contribute to underemployment and reduced quality of life. This scoping review mapped the contemporary evidence (2015–2025) on vocational training and employment‐related interventions for autistic adults, with particular attention to intervention types, strengths/limitations, professional roles, and contextual factors shaping implementation. Following the Arksey and O′Malley framework and PRISMA reporting guidance, we searched PubMed, MEDLINE and Web of Science in two stages (June 2024; updated November 2025), combining ASD terms with vocational rehabilitation/employment and training terms. Records were deduplicated and screened in Rayyan using predefined eligibility criteria (adults ≥ 18 years with ASD; English‐language empirical studies of vocational training interventions). The search identified 1117 records; after removing 434 duplicates, 683 records underwent title/abstract screening, 191 full texts were assessed, and 26 studies were included. Most studies were published from 2019 to 2025 (73.1%) and conducted in the United States (53.8%), followed by Japan (15.4%) and Australia (11.5%). Primary investigators most often represented psychology/psychiatry/clinical sciences (38.5%). Interventions were commonly delivered in workplace/community settings (57.7%), with highly variable dose: Among studies reporting calendar duration, the median was 23.0 weeks (range: 0.71–312), and among those reporting contact hours, the median was 32.0 h (range: 2–900). Qualitative synthesis identified three recurring themes: (1) integrated supported employment and work‐based learning pathways (e.g., internship‐to‐employment models, customized employment, and IPS‐informed approaches), (2) targeted work‐readiness and discrete skill acquisition interventions (notably job interview and workplace social communication training, including technology‐mediated formats), and (3) implementation context, stakeholder perspectives, and sustainability considerations. Overall, the evidence base is expanding but remains heterogeneous in intervention reporting and outcome measurement, underscoring the need for clearer specification of intervention components, professional roles, and occupationally meaningful outcomes.

## 1. Introduction

Adults with autism spectrum disorder (ASD) continue to experience significant obstacles in the job market, with studies indicating that only approximately 15% of this population secures full‐time employment [1, 2]. This stark disparity highlights the urgent need for effective vocational training interventions that address the unique barriers encountered by individuals with ASD. These barriers are multifaceted, encompassing difficulties with social communication, understanding workplace expectations, and navigating the often‐intimidating job interview process. Such challenges frequently cause underemployment, wherein individuals secure jobs that fail to utilize their full potential, ultimately diminishing their quality of life (QOL). Research on vocational training interventions for adults with ASD remains scarce, despite increasing awareness of the challenges they face. In particular, there is a lack of cross‐regional comparisons and limited involvement from professionals in this field.

Recent therapeutic techniques and technological advancements provide promising avenues for addressing these barriers. Virtual reality (VR), in particular, has emerged as a transformative tool for both vocational training and education, providing immersive and adaptable environments that allow individuals to develop critical skills with minimal risk [3, 4]. In educational contexts, VR can simulate complex scenarios, providing a unique platform for engagement and skill acquisition that surpasses traditional approaches [5]. For individuals with ASD, VR facilitates a safe space for developing social, communication, and task‐related competencies essential for educational and professional success [6, 7]. These applications underscore the potential of VR as an intersectional tool bridging vocational training and education, developing pathways for individuals with ASD to thrive in academic and workplace settings. Emerging interventions, including VR‐based job interview training and video modeling, have been investigated as prospective tools for improving vocational outcomes for individuals with ASD. VR interventions offer interactive and simulated training environments that mimic real‐world job situations, whereas video modeling provides structured visual demonstrations to facilitate skill acquisition [4, 8]. Despite these developments, significant gaps remain in the existing literature, particularly regarding regional differences in vocational training interventions and traditional support methods, professional involvement, and cultural attitudes toward employment support for individuals with ASD. Currently, little is known about how different professions contribute to vocational training, the specific methods they employ, and the challenges they encounter. To improve the overall quality of employment support and the education of professionals in this field, understanding these variations is crucial. Moreover, existing studies demonstrate substantial heterogeneity in research designs, implementation contexts, and outcome measures, making it difficult to synthesize consistent evidence or identify best practices [9]. Given this diversity and the emerging nature of the field, a *scoping review* represents the most appropriate methodological approach. Scoping reviews are designed to comprehensively map the range, scope, and nature of available evidence rather than to appraise quality or assess effect sizes [10, 11]. They are particularly useful for identifying knowledge gaps, clarifying key concepts, and informing future systematic reviews or empirical investigations [12]. Thus, this approach allows for a broader understanding of vocational training interventions across regions and professional contexts.

Through this review, we are aimed at exploring the effectiveness of vocational training interventions as well as the professional roles, strategies, and region‐specific challenges that shape these programs.

This scoping review is aimed at systematically exploring the breadth of the literature on vocational training interventions for adults with ASD. Through the identification and analysis of recently implemented vocational training approaches, including traditional job coaching and emerging technologies such as VR, we evaluate their effectiveness and limitations to guide future research and program development. Consequently, this review seeks to underscore gaps in the existing evidence, particularly in regions where research is scarce. Understanding these gaps is critical for fostering equitable access to vocational training and employment opportunities for adults with ASD worldwide. The findings from this review will inform the development of inclusive and effective vocational training programs that empower adults with ASD to achieve greater independence and fulfillment in their professional careers. This review contributes to the broader goal of enhancing the employability and overall well‐being of individuals with ASD by mapping current evidence and identifying areas for further investigation.

## 2. Materials and Methods

Scoping reviews are particularly effective in identifying the range of evidence, concepts, and gaps in research, especially in areas where the literature is complex and evolving [9]. To conduct this scoping review, we adhered to the five‐step framework proposed by Arksey and O′Malley [10]. This framework provides a structured and systematic approach for identifying, analyzing, and summarizing the relevant literature on vocational training interventions for adults with ASD. To further strengthen our process, we adhered to the Preferred Reporting Items for Systematic Reviews and Meta‐Analyses (PRISMA) guidelines [13], which offered a systematic framework for documenting our literature selection process and ensuring methodological rigor. Below, we describe each step in detail.

Step 1: Identify the research question

In any scoping review, formulating clear and focused research questions represents a crucial first step. Here, we are aimed at addressing the following broad research questions:

1. What vocational training interventions have been recently utilized to teach vocational skills to adults with ASD?
2. What are the strengths and limitations of these interventions?

These questions were intentionally broad to capture a broad range of interventions and their outcomes, ensuring that the review encompassed diverse perspectives and methodologies. Furthermore, this step established the scope of the review, defining its relevance to vocational training.

Step 2: Identify relevant studies

This step involved developing a comprehensive search strategy to identify studies addressing the review questions. The database search was conducted across three electronic databases—PubMed, MEDLINE (via EBSCOhost), and Web of Science Core Collection—in two stages. An initial search was undertaken in June 2024 to inform the scope and preliminary study identification. Following peer‐review feedback and to ensure the evidence base was current, we updated and expanded the search in November 2025 by incorporating additional keywords and database‐specific controlled vocabulary (e.g., MeSH terms and subject headings) and rerunning the full strategy across all databases. All records retrieved across both stages were exported, deduplicated, and screened using the same eligibility criteria.

The strategy combined two concept blocks using (1) ASD and related terms, and (2) vocational rehabilitation/employment and training‐related terms (including supported employment and common program labels). Database‐specific field tags and operators were applied (e.g., MeSH terms and [title/abstract] in PubMed; subject headings and subject mapping in EBSCOhost; proximity operators such as NEAR/x in Web of Science). Searches were limited to the most recent decade (2015–2025) to capture contemporary service models and employment contexts. Older studies may reflect outdated policies and workplace practices, so they may be less applicable today. To enhance completeness, reference lists of included studies and relevant reviews were hand‐searched, and citation searching was conducted where feasible (e.g., via Web of Science). A summary of the search is presented in Table 1, and the complete search strategies for each database are provided in Appendix A to support transparency and reproducibility.

**Table 1:** Databases and search strategies used.

Database (platform)	Search stage/date	Search strategy summary (details in appendix)	Limits applied
PubMed (MEDLINE)	June 2024 (initial) November 14, 2025 (updated)	Two concept blocks combined with (1) autism spectrum disorder terms and (2) vocational rehabilitation/employment/training terms. MeSH terms were combined with free‐text keywords using PubMed field tags (e.g., [MeSH Terms] and [Title/Abstract]) and Boolean operators (or/and). Examples of free‐text terms: autis∗, asperger∗, “pervasive developmental disorder∗”; supported employ∗, IPS, job placement, vocational training, work readiness, transition to work, and customized employ∗.	Publication date restricted to 2015–2025.
MEDLINE (via EBSCOhost)	June 2024 (initial) November 14, 2025 (updated)	Two concept blocks combined with (1) autism spectrum disorder terms and (2) vocational rehabilitation/employment/training terms. Database subject headings (e.g., MH/MM) were combined with free‐text keyword searching (TI/AB and relevant field tags), using Boolean operators (OR/AND). Subject heading mapping was enabled where supported.	Publication date restricted to 2015–2025.
Web of Science Core Collection	June 2024 (initial) November 15, 2025 (updated)	Two concept blocks combined with (1) autism spectrum disorder terms and (2) vocational rehabilitation/employment/training terms. Free‐text searching was conducted in title/abstract fields using Boolean operators (or/and) and proximity operators (e.g., NEAR/x) where appropriate. Examples of terms: autis∗, “autism spectrum disorder”, asperger∗; supported employ∗, IPS, job placement, vocational training, work readiness, and transition to work.	Publication years restricted to 2015–2025.

*Note:* The search was conducted in two stages (initial exploratory search in June 2024; updated and expanded search in November 2025). Full database‐specific search strategies and complete line‐by‐line syntax are provided in Appendix A.

Step 3: Study selection

In this step, we systematically screened the studies identified in the previous stage. The selection process involved the following three phases:

1. Title screening: Titles were reviewed for relevance to the research questions.
2. Abstract screening: Abstracts of the remaining studies were examined to determine their alignment with the inclusion criteria.
3. Full‐text screening: Full‐text articles were assessed to confirm eligibility.

The following were the inclusion criteria:

‐ Participants aged ≥ 18 years with a primary diagnosis of ASD

‐ Articles published in English

‐ Studies employing quantitative, qualitative, mixed‐methods, or exploratory designs

‐ Interventions focused on vocational training skills

The following were the exclusion criteria:

‐ Participants aged < 18 years

‐ A primary diagnosis other than ASD

‐ Studies with no full text available

‐ Gray literature (e.g., nonreviewed articles, theses, or magazine articles)

‐ Pharmacotherapy‐focused studies

Duplicates were removed; to ensure consistency during the selection process, a team‐based approach was employed. Discrepancies between the reviewers were resolved through discussion or consultation with a third researcher, ensuring rigor and transparency in the study selection.

Step 4: Chart the data

Data charting entailed extracting and organizing relevant information from the included studies. To ensure consistency, a standardized data extraction table was developed. The table captures the following key study characteristics:

‐ Title

‐ Author and publication year

‐ Main author′s discipline

‐ Sample size and country of study

‐ Study design and purpose

‐ Participant demographics (e.g., age and diagnosis)

‐ Intervention details (e.g., type of vocational training, setting, and intervention duration)

‐ Evaluation tools employed for assessing outcomes

‐ Key results and limitations or challenges

To ensure accuracy and reliability, each researcher independently extracted the data. Discrepancies were resolved collaboratively. This step facilitated the systematic organization of data, facilitating clear identification of themes and trends across studies.

Step 5: Collate, summarize, and report the results

The final step involved synthesizing the findings to provide a comprehensive overview of vocational training interventions for adults with ASD. Results were narratively summarized and, where possible, supported with tables and figures. This synthesis identified key trends and gaps related to the types of interventions used, the settings in which interventions were delivered, and the tools used to evaluate outcomes. We also highlighted gaps in the literature, particularly in underrepresented regions, to address the research questions and provide actionable insights for occupational therapy (OT) practice.

## 3. Conclusion of the Framework

By adhering to the Arksey and O′Malley framework [10], this scoping review systematically mapped the current literature and identified key interventions, their outcomes, and research gaps. By employing a structured approach, we ensured the transparency, reproducibility, and relevance of our findings, which are aimed at guiding future research and program development in vocational training for adults with ASD.

## 4. Results

**Figure 1:**
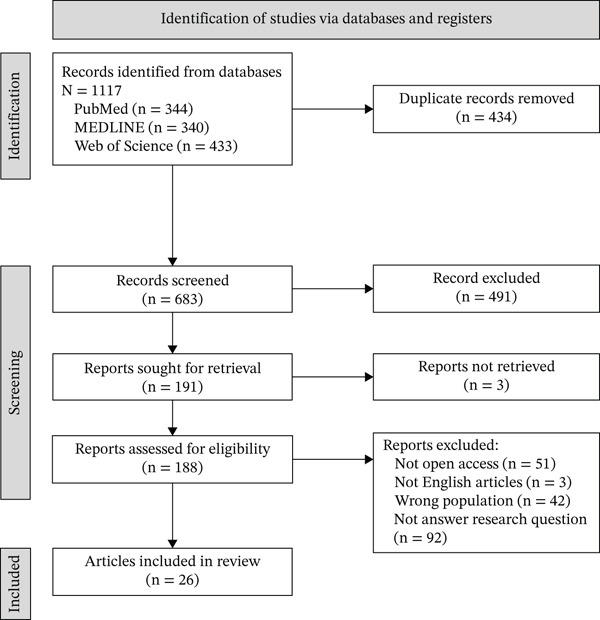
Study selection process.

Using the PRISMA guidelines (Figure 1), the search process identified 1117 records. After removing 434 duplicates and completing database/import checks, 683 records proceeded to title and abstract screening in Rayyan systematic review software. Following this screening stage, 191 full‐text articles were assessed for eligibility, and 26 studies met the inclusion criteria for the final synthesis.

The screening process was conducted in three sequential phases (title, abstract, and full‐text screening). H.A.H. and D.F. independently screened records against predefined inclusion criteria aligned with population, conceptual, and cultural considerations (PCC). To ensure comprehensive coverage, search terms were iteratively refined during the screening process. A team‐based approach consistent with guidance by Levac et al. [11] was used to maintain methodological rigor. Discrepancies were resolved through reviewer discussion, with R.M. serving as the final adjudicator when consensus could not be reached.

### 4.1. Quantitative Results

Quantitative data were summarized using descriptive statistics (frequencies and percentages for categorical variables; means and standard deviations or medians with interquartile ranges and ranges for continuous variables). A descriptive statistic could be the value of a single data point (variable) or a summary of one or more variables. Descriptive statistics typically include data distributions [14]. The 26 included studies varied in study design, geographic location, participant characteristics, intervention dose/duration, outcome measurement approaches, and the disciplinary backgrounds of the primary investigators. Table 2 shows the characteristics of included studies.

**Table 2:** Characteristics of included studies (*n* = 26).

Study	Country or region	Main author disciplines	Study design	Participants (N, age, diagnosis)	Settings
Schall et al. 2015[15]	United States	Vocational rehabilitation; special education	Retrospective observational clinical records review	*N* = 45; ASD	Community‐based
Wehman et al. 2016[16]	United States	Rehabilitation/vocational rehab	RCT	*N* = 64; age 19–59 years; ASD	Research laboratory; workplace/community employment context
Kumazaki et al. 2017[17]	Japan	Psychiatry and neuroscience, interdisciplinary with assistive robotics and rehabilitation technology	RCT	*N* = 15; age 18–25 years; ASD	Workplace/community employment context
McLaren et al. 2017[18]	United States	Child and adolescent psychiatry/ASD clinical services	Pilot study	*N* = 5; age 19–28 years; ASD	Community
Baker‐Ericzén et al. 2017[19]	United States	Clinical	Pilot study	*N* = 8; age 18–29 years; ASD	Community
Nadig et al. 2018[20]	Canada	Psychology/psychiatry	RCT	*N* = 26; age 18–32 years; ASD	Clinic
Walsh et al. 2017[21]	Ireland	Psychology/ABA	Multiple‐probe single‐case design	*N* = 7; age 19–22 years; ASD + ID	Vocational rehabilitation‐training center
Flower et al. 2019[22]	Australia	Business/management; psychology/psychiatry	Qualitative exploratory design	*N* = 6; mean 38.6 years; ASD + stakeholders	Workplace/community employment context
Grob et al. 2018[23]	Japan	Applied behavior analysis	Single‐case design	*N* = 3; age 19–27 years; ASD	Workplace/community employment context; University setting
Honsberger et al. 2018[24]	United States	Education	Single‐case experimental design: multiple probe design across tasks (for each participant), with baseline → intervention → follow‐up conditions.	*N* = 4; age 19–22 years; ASD	School‐based food truck
Kumazaki et al. 2019[25]	Japan	Clinical/medical + developmental/child mental health	RCT	*N* = 29; age 18–27 years; ASD	Workplace/community employment context
Wehman et al. 2019[26]	United States	Vocational rehabilitation	RCT	*N* = 156; age 18–21 years; ASD	Hospital
Di Sarro et al. 2020	Italy	Mental health and disability services	Descriptive observational case‐series design	*N* = 5; age 18–42 years; ASD	Research laboratory; workplace/community employment context
Gorenstein et al. 2020[27]	United States	Psychiatry	RCT	*N* = 22; age 18–45 years; ASD	Community
Roberts et al. 2020[28]	United States	ABA	Single‐case experimental design	*N* = 3; age 19–20 years; ASD	School
Whittenburg et al. 2019[29]	United States	Vocational rehabilitation	RCT	*N* = 14; age 18–21 years; ASD	Army installation
Peijen et al. 2021	Netherlands	Public policy staff	Quasi‐experimental	*N* = 38; mean 27.26 years; ASD	Community employment
Hayward et al. 2022[30]	Australia	Psychology	Mixed methods	*N* = 84; ASD + stakeholders	NR
Kumazaki et al. 2022[31]	Japan	Psychiatry and neuroscience, interdisciplinary with assistive robotics and rehabilitation technology	Single‐case design	*N* = 15; age 18–24 years; ASD	Clinic
Moody et al. 2022[32]	United States	Psychology/psychiatry	RCT	*N* = 12; age 19–30 years; ASD	Workplace/community employment context
Brighenti et al. 2023[33]	Italy	Psychology/psychiatry	Experimental study	*N* = 10; age 21–37 years; ASD	Workplace/community employment context
Kahng et al. 2023[34]	United States	Behavior analysis; psychology/psychiatry	RCT	*N* = 7; age 20–28 years; ASD	Workplace/community employment context; community
Wehman et al. 2022[35]	United States	Vocational rehabilitation	RCT	*N* = 37; age 18–21 years; ASD	Military base implementation of PS + ASD
Ashburner et al. 2024[36]	Australia	Psychologist	Qualitative evaluation using semistructured interviews	*N* = 26; age 19–41 years; ASD + stakeholders	NR
Lousky et al. 2024	United States	Psychology/psychiatry	RCT	*N* = 49; age 18–23 years; ASD	Community; clinic
Randolph et al. 2025[37]	United States	Special education	Qualitative study using semistructured interviews	*N* = 16; age 21–40 years; ASD	NR

*Note:* Discipline and setting categories reflect reporting in the included studies.

Abbreviations: ASD, autism spectrum disorder; ID, intellectual disability.

#### 4.1.1. Publication Year and Geographic Distribution

The included studies were published between 2015 and 2025, with most published from 2019 to 2025 (*n* = 19; 73.1%). The largest number of studies were published in 2019 (*n* = 5; 19.2%) and 2020 (*n* = 4; 15.4%). Studies were conducted primarily in the United States (*n* = 14; 53.8%), followed by Japan (*n* = 4; 15.4%) and Australia (*n* = 3; 11.5%). Additional studies were conducted in Italy (*n* = 2; 7.7%), and Canada, Ireland, and the Netherlands (each *n* = 1; 3.8%).

#### 4.1.2. Disciplinary Background of Primary Investigators

Primary investigators most frequently represented psychology/psychiatry/clinical sciences (*n* = 10; 38.5%), followed by rehabilitation/vocational fields (*n* = 5; 19.2%), ABA/behavior analysis (*n* = 4; 15.4%), and education/special education (*n* = 3; 11.5%). Interdisciplinary/technology backgrounds were identified in two studies (7.7%), and business/management and public policy were each represented by one study (3.8%).

#### 4.1.3. Study Design

Randomized controlled trials were the most common design (*n* = 11; 42.3%), followed by single‐case experimental designs (*n* = 5; 19.2%) and qualitative studies (*n* = 3; 11.5%). Observational and pilot designs were each represented by two studies (7.7%), whereas quasi‐experimental, mixed methods, and nonrandomized experimental designs were each represented by one study (3.8%).

#### 4.1.4. Participant Characteristics (Sample Size, Age, Diagnosis)

Across the 26 studies, the total sample size was 715 participants, with individual study sample sizes ranging from 3 to 156. The mean sample size was 27.5 (SD: 33.1) and the median was 15 (IQR: 27.75). By sample size category, 11–30 participants were most common (*n* = 10; 38.5%), followed by ≤ 10 participants (*n* = 9; 34.6%) and > 30 participants (*n* = 7; 26.9%).

All studies reported age (26/26; 100%). Across studies, participants ranged from 18 to 59 years. Using a study‐level midpoint (range midpoint when a range was reported; mean age when only a mean was reported), the median age midpoint was 23.5 years (IQR: 8.5).

Most studies included participants with ASD only (*n* = 22; 84.6%). Three studies included ASD plus stakeholders (e.g., family members, employers, and coworkers; *n* = 3; 11.5%), and one study included ASD with co‐occurring intellectual disability (*n* = 1; 3.8%).

#### 4.1.5. Settings

Settings were most frequently workplace/community‐based (*n* = 15; 57.7%), followed by other/unspecified settings (*n* = 5; 19.2%), clinical/healthcare settings (*n* = 4; 15.4%), and educational settings (*n* = 3; 11.5%). Smaller numbers of studies were conducted in research/laboratory settings (*n* = 2; 7.7%) and vocational rehabilitation/training centers (*n* = 1; 3.8%). (Setting categories were not mutually exclusive when multiple settings were reported.)

#### 4.1.6. Intervention Duration and Intensity

Sixteen studies (16/26; 61.5%) reported intervention duration in a format that could be expressed as calendar time (days/weeks/months/years). Among these, intervention duration was highly variable, with a median of 23.0 weeks (IQR: 29.1), ranging from 0.71 to 312 weeks.

Nine studies (9/26; 34.6%) explicitly reported contact time in hours, with a median of 32.0 h (IQR: 32.5) and a wide range (2–900 h). The remaining studies described intervention exposure primarily using session‐based or mastery‐based formats that could not be consistently converted into total duration or total hours.

#### 4.1.7. Outcome Measurement Approaches

Outcome measurement most frequently relied on self‐report questionnaires/scales (*n* = 18; 69.2%) and employment/vocational outcomes (*n* = 15; 57.7%). Qualitative methods (e.g., interviews and focus groups) were also common (*n* = 12; 46.2%). Measures of ASD traits/symptoms were used in 11 studies (42.3%), and cognitive/intellectual assessments in nine studies (34.6%). Observed performance/probe measures were reported in five studies (19.2%), and fidelity/implementation measures in three studies (11.5%).

### 4.2. Qualitative Results

Content analysis is a systematic method for document analysis used to describe and quantify events, investigate theoretical difficulties, and refine words into fewer content‐related categories. This approach assumes that words, sentences, and other similar elements have the same meaning when aggregated [38]. In this review, a descriptive content analysis was conducted to identify recurring patterns and concepts across the included studies. The analysis followed the procedures described by Elo and Kyngäs in 2008, who present qualitative content analysis as a structured approach that can be conducted inductively (deriving categories directly from the data) or deductively (applying an existing framework). In the preparation phase, researchers immerse themselves in the data through repeated reading and determine the unit of analysis (e.g., words, phrases, or sentences). In the organization phase, relevant meaning units are coded and then grouped into subcategories and broader categories through abstraction (or mapped onto a categorization matrix in a deductive approach). Finally, in the reporting phase, the resulting category structure is described clearly and supported with representative examples to demonstrate how the findings address the research aims [38]. During the preparation phase, all included articles were read multiple times to ensure familiarity with the data. In the organization phase, meaningful units of text (e.g., phrases or sentences) related to vocational training for adults with ASD were extracted and coded manually. Codes with similar meanings were grouped into subcategories and then abstracted into broader categories through an inductive process.

To enhance rigor, two researchers independently conducted the coding and met to compare and refine the categories through discussion until consensus was reached. Any disagreements were resolved collaboratively to ensure credibility and consistency. Qualitative data management was performed using Microsoft Excel rather than specialized software, given the manageable number of included studies. The descriptive content analysis identified three overarching themes that characterize the intervention landscape and evaluation emphasis across the included studies. Each theme is supported by multiple studies and reflects recurrent patterns in intervention design, target outcomes, and contextual determinants of success. The qualitative findings are summarized in Table 3..

**Table 3:** Intervention characteristics, evaluation tools, and key findings.

Study	Purposes	Intervention terms	Tasks	Evaluation tool	Results
Schall et al. 2015[15]	To compare supported employment (SE) outcomes with versus without prior Project SEARCH+ autism spectrum disorder (ASD) supports (PS + ASD), focusing on (1) time spent in SE phases (JD, JST, and LTS), (2) job retention at 6/12/18 months, and (3) wages earned.	PS − ASD + SE group: Project SEARCH = 9 months (~900 hours) + supported employment after = ~312 hours (JD 80.64 + JST 161.85 + LTS 69.22). SE‐only group: Supported employment total = ~351 h (JD 184.00 + JST 117.06 + LTS 49.50).	Intervention is a service model rather than a single standardized task. SE phases include job seeker profile, job development, job site training (systematic instruction for job skills/work behaviors), and long‐term supports (follow‐along monitoring/support; can increase again if needs change). PS‐ASD includes participation in internships within a host business plus classroom instruction and ASD‐specific supports (ABA‐based systematic instruction, autism/behavior specialist consultation, and staff training).	Extracted from clinical records (intake forms, hourly billing forms, employment notes, and outcome records). Outcomes: hours of intervention in SE phases (JD/JST/LTS), wages earned, jobs acquired, and job retention at 6/12/18 months.	SE yielded competitive employment outcomes for this sample; compared with SE‐only, the PS − ASD + SE group required significantly fewer job development hours (80.64 vs. 184.00; *p* = 0.007), earned a higher mean hourly wage ($9.89 vs. $8.82; *p* = 0.011), and demonstrated higher job retention at 12 and 18 months (84% vs. 55%; *p* = 0.033), with a trend at 6 months (96% vs. 75%; *p* = 0.052). Most participants were employed part‐time in the most recent job record (88.9%).
Wehman et al. 2016[16]	The purpose of this study was to examine the effectiveness of supported and customized employment services in achieving competitive integrated employment (CIE) outcomes for young adults with ASD, through a retrospective review of individuals served by a vocational rehabilitation program.	6 years	Participation in supported and customized employment activities across four intervention Phase 1. Discovery and situational assessment ∗ Interviews, observations, community exploration ∗ Short‐term work trials in real workplaces 2. Job development and career search ∗ Working interviews ∗ Job matching and job customization ∗ Interview preparation and role‐play 3. Job‐site training and support ∗ Learning job tasks in real workplaces ∗ Use of visual schedules, reminders, scripts ∗ Applied behavior analysis (ABA)–based instruction ∗ Assistive technology (e.g., iPod Touch) 4. Long‐term support and job retention ∗ Fading of supports ∗ Ongoing monitoring and problem‐solving ∗ Coordination with employers and community supports	Primary evaluation is conducted using the AIR Self‐Determination Scale. Secondary evaluation tools include the Career Development Inventory–Australia, the Personal Wellbeing Index–School Children, the Learning Climate Questionnaire, the Transition Planning Objectives Scale, and the Social Responsiveness Scale–Second Edition.	Nearly all participants (98.4%) achieved CIE, with the majority securing customized job placements. Intervention intensity decreased substantially over time, with employment stabilization typically achieved by Week 7.
Kumazaki et al. 2017[17]	The study is aimed at examining the feasibility and preliminary efficacy of an android robot–mediated mock job interview intervention for young adults with ASD, specifically focusing on changes in self‐confidence and physiological stress responses compared with a self‐study control condition.	Three consecutive days, with participants completing one 10‐min android robot–mediated mock job interview per day.	participants first completed a mock job application and a baseline mock job interview with a human interviewer. During the intervention phase, the android robot group completed three consecutive days of 10‐min mock job interviews with the robot, whereas the control group engaged in independent study of interview‐related materials. All participants completed human‐conducted mock job interviews at baseline and postintervention, followed by self‐confidence ratings and saliva collection.	Self‐confidence was measured using a Likert‐scale self‐report confidence rating following mock interviews. Physiological stress was assessed using salivary cortisol levels collected after interview sessions. Additional baseline measures included the Autism Spectrum Quotient–Japanese version, the Liebowitz Social Anxiety Scale, and standardized intelligence testing.	All participants completed the study without adverse events, demonstrating feasibility and acceptability of the intervention. The android robot–mediated group showed significantly lower salivary cortisol levels over time compared with the control group, indicating reduced physiological stress. Self‐reported interview confidence showed a trend toward improvement in the robot group, although this did not reach statistical significance due to the small sample size. Overall, the findings provide preliminary evidence that robot‐mediated mock interviews may help reduce anxiety and support confidence during job interview preparation for young adults with ASD.
McLaren et al. 2017[18]	To describe a pilot IPS supported employment program adapted for young adults with ASD and to evaluate outcomes for the first five participants over 1 service year (competitive employment and broader functional/psychosocial changes).	Total duration: 1 year of IPS supported employment services per participant (“1 service year”). Frequency/dose: Individualized (not fixed schedule reported); delivered by an employment specialist (program staffing: 0.5 FTE = 20 h/week). Time to employment (indicator of intensity): Participants obtained competitive jobs within 1–4 months of program entry.	IPS core activities: Rapid job search, preference‐based job matching, interview/job acquisition support, and ongoing supports (including maintaining employer contact; additional support during mental health symptom exacerbations), plus coordination with mental health providers and high parent involvement.	Competitive employment outcomes (job obtained, time to job, job type, wages; described in Table 1); qualitative feedback/observations from participants, parents, employers; baseline SRS‐2 parent rating; program fidelity assessed using the IPS Supported Employment Fidelity Scale.	Over 1 year of IPS, all five participants obtained competitive employment (within 1–4 months), including placements described as competitive/high‐demand for some. Reported improvements included increased pay/work hours, improved hygiene, self‐esteem, social relationships, employment satisfaction, and greater independence (e.g., transportation, moving toward living independently). Parents and clinicians reported improvements in mental health and reduced severity of comorbid symptoms; employers reported positive satisfaction and performance.
Baker‐Ericzén et al. 2017[19]	To test the feasibility, acceptability, satisfaction, and initial outcome signals of a newly developed manualized “soft skills” intervention (SUCCESS) embedded in a supported employment/vocational training setting, targeting executive functioning and social cognition/communication in adults on the autism spectrum.	Intervention length (dose): 6 months total, delivered as 25 weekly group sessions, 1.5 h/session (≈37.5 h of SUCCESS intervention)	Manualized group curriculum covering cognitive enhancement (13 sessions) then social cognition/social skills (12 sessions) with psychoeducation, strategy teaching, experiential activities, discussion, application activities, and weekly practice assignments (“try it and apply it”). Performance tasks included vocational role‐plays such as “water cooler” coworker conversation and requesting time off from a supervisor (SSPA 2.0).	Executive functioning: D‐KEFS (performance‐based) and BRIEF‐A (participant + parent report). Social cognition/communication: SRS‐2 (participant + parent report) and SSPA 2.0 (employment version) with vocational role‐play scenes (video‐coded; ICC reported). Daily living/vocational: Functional Daily Living Questionnaire; employment interview (jobs/hours/wages).	Feasibility/acceptability: 25 sessions delivered to fidelity; 8/9 completed; attendance and in‐session work completion high; homework completion high; satisfaction high. Outcomes: Improvements reported on multiple executive‐function and social measures (self‐report BRIEF‐A improvements; D‐KEFS improvements on several domains; SRS‐2 improvements in selected domains; SSPA vocational role‐plays improved on multiple rated dimensions). Employment: Proportion employed increased from 22% to 56% postintervention; mean weekly work hours among those employed increased (reported from 6 h/week to ~20 h/week), with competitive wages reported.
Nadig et al. 2018[20]	The purpose of the study was to evaluate the effectiveness of a brief group‐based transition support program for adults with ASD without intellectual disability, specifically examining its effects on self‐determination, quality of life, and social problem‐solving during the transition to adulthood.	10 weeks	Participants attended 10 weekly group sessions lasting 2 h each. Sessions focused on social communication skills, self‐determination strategies such as goal setting and self‐advocacy, and collaborative skills for working with others. Curriculum modules were selected based on participants′ self‐identified needs, and activities included guided discussion, problem‐solving exercises, role‐based scenarios, and structured reflection.	Primary evaluation tools included the ARC Self‐Determination Scale, an abridged version of the quality of life questionnaire, and a Social Problem‐Solving Task. Additional measures included needs assessment questionnaires, participant and parent skill ratings, and follow‐up questionnaires at 1 year.	The intervention produced positive effects on quality of life and self‐determination. Participants in the intervention group reported higher quality of life scores compared with the waitlist control group and showed significant improvement in the interpersonal cognitive problem‐solving subdomain of self‐determination. Participants also rated their own skills in targeted domains significantly higher after the intervention, and parent ratings confirmed these improvements. No significant intervention effect was found for performance on the social problem‐solving task. Follow‐up data suggested that some gains in self‐determination and quality of life were maintained 1 year after program completion.
Walsh et al. 2017[21]	To evaluate the effectiveness of Walker Social Skills Curriculum: ACCESS program plus video modeling to increase social communication skills necessary for workplace inclusion in adults with ASD + intellectual disability, including changes in social skills and problem behaviors, with maintenance/generalization.	20 weeks total; 2 sessions/week (Monday and Wednesday); 1.5 h/session = 3 h/week → ~60 h total intervention contact time (group‐based).	ACCESS curriculum instruction across three domains: peer‐related, adult‐related, self‐related social skills (31 lessons; 30 target skills in Lessons 2–31). Instruction used video models (positive/negative examples), discrimination practice, role‐plays (multiple trials), feedback with prompting, and generalization planning via student contracts; probes embedded in daily center activities (e.g., greeting, conversations, responding to requests, work habits, and self‐control/organization).	Direct observational performance probes of target social skills (occurrence/nonoccurrence) across natural center routines; SRS‐2 (teacher/parent ratings), SSiS‐RS (teacher ratings: social skills and problem behaviors), ACCESS placement test (teacher‐rated), plus 3‐month follow‐up probes; treatment fidelity and interobserver agreement recorded; social validity questionnaires (participants/parents/teachers).	Direct probes: Baseline low performance (8%–41%), increased postteaching (73%–100%) and maintained at 3‐month follow‐up (73%–100%). SSiS: significant increase in social skills standard scores and significant decrease in problem behaviors. SRS‐2: total score decreased but not significantly. ACCESS placement test: significant improvements in total and subscales (peer/adult/self). Social validity positive. Some real‐world indicators reported (e.g., one participant obtained employment; others attended interviews; one gained community work experience access postintervention), but authors caution these cannot be attributed solely to the intervention.
Flower et al. 2019[31]	The main purpose of the study was to examine an autism‐specific employment program as an alternative pathway to employment by exploring the experiences of autistic employees and their coworkers, and by evaluating the perceived benefits, barriers, and outcomes of an extended onsite training and assessment hiring model.	A 3‐week paid, full‐time onsite training and assessment period, followed by a transition‐to‐work phase with ongoing job‐coach support during the initial months of employment.	The intervention was an autism‐specific employment program known as the Rise@DHHS program, which incorporated an extended onsite vocational training and assessment model delivered in collaboration with Specialisterne Australia. Participants completed a 3‐week paid, full‐time training and assessment program conducted onsite at the employer organization, followed by transition‐to‐work training and ongoing job‐coach support during the first months of employment. Tasks included simulated and real job duties, workplace orientation, role‐specific training, autism awareness training for coworkers, and individualized workplace adjustments.	Evaluation was qualitative in nature and included semistructured one‐on‐one interviews with autistic employees and semistructured focus groups with their nonautistic coworkers. Data were analyzed using inductive content analysis supported by NVivo software.	The findings indicated that the extended training and assessment process reduced traditional recruitment barriers, improved role preparedness, and supported smoother transitions into employment for autistic employees. Participants reported increased workplace confidence, improved well‐being, greater independence, and stronger social integration. Coworkers reported improved understanding of autism and positive attitudes toward inclusive employment, although some perceived the program as preferential treatment. Overall, the results suggest that onsite vocational training and assessment programs can be an effective alternative pathway to competitive employment for autistic adults.
Grob et al. 2018[23]	The purpose of the study was to assess and teach job‐related social skills to adults with ASD using behavioral skills training (BST) and stimulus prompts, and to evaluate whether these interventions improved appropriate social responding in simulated workplace situations and generalized across settings and supervisors.	2 h variable; training continued until mastery criteria were achieved.	Participants completed entry‐level office and retail‐type tasks designed to evoke job‐related social behaviors. These tasks included folding or hanging clothing, filing papers, sorting items, stuffing envelopes, stapling, shredding documents, data entry, stocking shelves, counting money, cleaning, and computer‐based tasks. Supervisors intentionally created situations such as vague instructions, missing materials, and corrective feedback to assess social skill responses.	Job‐related social skills were evaluated using direct behavioral observation during structured simulated work sessions. Observers recorded the percentage of correct responses across predefined opportunities for each target skill. Interobserver agreement and procedural integrity were calculated to ensure reliability and fidelity of measurement.	The intervention was effective in increasing job‐related social skills for all three participants. Skills such as making confirming statements, asking for help or task models, apologizing, and responding appropriately to feedback improved substantially following BST with stimulus prompts. Maintenance of skills was observed over time, and generalization to a novel supervisor and setting occurred, although some participants required additional supports such as multiple exemplar training for consistent generalization. Overall, the findings demonstrated that BST combined with stimulus prompts is a feasible and effective approach for preparing adults with autism for workplace social demands.
Honsberger et al. 2018[24]	To examine whether a peer‐mediated literacy‐based behavioral intervention (LBBI) used as a job coaching strategy would (1) increase acquisition of three work skills comprising an employment routine for secondary students with ASD and (2) whether skills would maintain after LBBI removal.	Duration: session‐based; peer training 40 min total; student intervention continued until mastery, typically 5–10 sessions per task (Beth: 22 sessions; Meg: 25 sessions; Gwen: Task 2–3 = 10 sessions; Task 1 session count not explicitly stated).	Three vocational tasks in a coffee‐service routine: (1) setting up the food truck, (2) setting up the coffee service, (3) filling a coffee order (barista)—each broken into 13–15 steps (task analysis table provided in the article).	Primary outcome: number/percentage of correct independent steps on task analyses for each job skill (live observation; paper–pencil data sheets). Interobserver agreement collected for a subset of sessions (overall high agreement reported). Post hoc effect size: percent of nonoverlapping data (PND). Social validity: short questionnaires completed by staff and students. Participant characterization tools also reported (e.g., ADOS‐2, CARS‐2HF, WAIS‐4, DAR‐2, Differential Abilities Scale).	Across all three trainees, baseline performance was near 0% independent correct steps, then increased rapidly to high accuracy (typically ~93%–100%) during intervention for each task, and maintained during follow‐up after LBBIs were removed, reported PND = 100*%* for baseline‐to‐intervention and baseline‐to‐follow‐up across all skills/students. Social validity responses indicated staff and students viewed the approach as acceptable/feasible and valued the job skills taught.
Kumazaki et al. 2019[25]	To evaluate whether a job interview training program using an android robot (JUA)—targeting nonverbal communication—improves job interview nonverbal performance, self‐confidence, and stress (salivary cortisol), compared with interview guidance by teachers (IGT) alone.	IGT‐only group: ~30 min/day × 5 days = 150 min total (2.5 h) IGT + JUA group: ~(30 min IGT + 30 min JUA) = ~60 min/day × 5 days = 300 min total (5 h)	Mock job interview tasks: Participants selected a job (from six options) and answered standardized interview questions about themselves, reasons for applying, expertise, disability, weaknesses, duties, and so forth. JUA tasks: (1) teleoperate the android and converse via the robot; (2) face‐to‐face mock interview conducted by the android robot; (3) feedback + nonverbal communication exercises using the android robot. Outcome assessment task: mock job interview with a human interviewer (≈20 min) using a scripted protocol.	Nonverbal communication performance during human mock job interview (Days 1 and 7): 7‐point Likert ratings (0–6) across posture, gaze, voice volume, nodding, facial expressions; primary rater blinded; reliability reported (ICC = 0.91). Self‐confidence rating scale after human interviewer sessions (0–6). Physiological stress: salivary cortisol collected at S1: baseline, S2: immediately postinterview, S3: 20 min, S4: 40 min (Days 1 and 7), with change ratios (e.g., S4/S1). Participant characterization measures included AQ‐J, WAIS‐IV IQ, and LSAS.	Compared with IGT alone, IGT + JUA produced significantly greater postintervention improvements in all rated nonverbal domains (posture, gaze, voice volume, nodding, and facial expressions), higher self‐confidence, and greater reduction in stress indicators (significant group difference in S4/S1 cortisol change; trend for S3/S1). All combined‐group participants completed the program and all answered “yes” to wanting the intervention again; in IGT‐only, three dropped out and 53.8% said “yes.” Kumazaki et al. 2019
Wehman et al. 2019[26]	To replicate and expand prior trial findings by testing whether PS + ASD improves CIE outcomes for 18–21‐year‐old students with ASD receiving public special education services, compared with business‐as‐usual high school services.	Total duration: 9 months (entire school year). Frequency/intensity: 35 h/week of community‐based employment training (CBET).	Treatment: participation in hospital‐based internship work tasks tailored to strengths/interests and progressively refined across three rotations (examples given include data entry/scanning/database tasks, janitorial/cleaning, stocking/supply tasks, etc.), plus structured employability activities (e.g., resume development, internship presentations using photos/videos, planning meetings, “working interviews,” video resumes, recommendation letters). Control: typical high school academic/nonacademic coursework and IEP services, with limited vocational coursework.	Vocational Index for Adults with ASDs (employment status at graduation and 1‐year follow‐up). Support Intensity Scale (SIS) Social Responsiveness Scale–2 (SRS‐2)	CIE outcomes were substantially higher in PS + ASD. At graduation: 32% (25/79) treatment employed vs 5% (2/42) control. At 1‐year: 73.4% (58/79) treatment employed vs 17% (4/24) control (for those with control data). Employed treatment participants worked about 21.2 h/week at ~$9.61–$9.67/hour at 1‐year. Mean time from graduation to employment was 18.8 weeks (treatment) vs 43.4 weeks (control, with 52 weeks assigned to those still unemployed at 1‐year). Among treatment participants who obtained employment during the study (64/79), retention at 1‐year was 90.6% (58/64). Jobs spanned multiple industries (healthcare, foodservice, retail, hospitality, distribution, etc.).
Di Sarro et al. 2020	The purpose of the study was to describe and evaluate an innovative employment access project for young adults with high‐functioning ASD, implemented through a collaboration between a public health autism service and a private company, with the aim of facilitating successful entry into competitive employment.	6 months	Participants completed a 6‐month paid internship, totaling 560 h, with the possibility of renewal for an additional 6 months. Tasks included participation in real workplace activities, social skills group training, individual and group support meetings, job coaching in the workplace, cognitive and functional assessments, and structured feedback sessions with clinicians, job coaches, and the employer. The training emphasized observation, modeling, practice, and immediate feedback.	Evaluation relied on clinical interviews, cognitive assessments, including WAIS‐IV or equivalent tools, the Assessment of Functional Living Skills (AFLS) for social and work‐related skills, and ongoing monitoring by clinicians and job coaches during the internship.	The project demonstrated that the BST‐based supported employment model was effective in small groups, facilitating learning through observation and modeling and improving social and professional skills relevant to the workplace. Participants showed increased autonomy, improved social functioning, and successful integration into a real work environment. The collaboration also increased employer awareness of the strengths and potential of individuals with autism, supporting the feasibility of inclusive employment pathways.
Gorenstein et al. 2020[27]	Pilot evaluation of a brief behavioral, employment‐related social skills intervention for adults with ASD in a community setting.	Total length: 15 weeks (group‐delivered). Frequency: Weekly sessions are implied (table lists “weekly session topics” across the program). Per‐session duration (minutes/hours): not clearly stated in the intervention description (no explicit minutes/hours reported). Follow‐up: employment/skills questionnaire at 6 months posttreatment.	Group discussions, practice exercises (including role‐play and video self‐modeling), worksheets, and homework assignments.	Social Responsiveness Scale‐2 (SRS‐2) self‐report + relative‐report Reading the Mind in the Eyes Test (RMET).	Significant improvement in caregiver/relative‐reported SRS‐2 social cognition and social communication subscales for the treatment group. No significant effects found on self‐report measures. At 6‐month posttreatment, ~45% reported an employment gain or increase; participants reported high satisfaction and perceived benefit.
Roberts et al. 2020[28]	To replicate/extend prior BST interview‐training work (Stocco et al. 2017) by testing whether BST improves three interview skill domains in young adults with ASD: answering questions, asking questions, and appropriate body language, including generalization and maintenance, plus social validity.	BST program completed within ~4 months (including school breaks). Schedule: 3 sessions/week per participant; included ~1 generalization probe/week. Session duration: baseline/interview probes ~5–15‐min; BST training sessions ~15–30 min.	Simulated job interviews using 10 common interview questions (asked in varied order/wording). Participants trained to: (a) give a direct answer + added detail; (b) pause then ask job‐relevant questions; (c) display appropriate posture/orientation/stillness. BST components: instructions + modeling + role‐play + feedback/rehearsal. For one participant, added textual prompts + token reinforcement.	Direct observation Checklist of treatment integrity 7‐point Likert questionnaire for social validity	All three participants improved over baseline across the three interview skill domains with BST. BST alone was sufficient for two participants; one participant required added textual prompts + token system + reinforcement to reach criterion. Maintenance generally strong through 6 weeks, with one booster for Sara′s body language at 6 weeks. Social validity ratings improved substantially from baseline to maintenance (mean ratings increased from ~2.3 to ~5.6 on 7‐point scales). The paper also notes real‐world vocational progress within 6 months (e.g., job offers/employment/internship changes), described descriptively rather than as a controlled outcome.
Whittenburg et al. 2019[29]	To report preliminary (Year 1) outcomes from a randomized waitlist‐controlled trial examining the efficacy of PS + ASD for military‐dependent/‐connected youth with ASD, focused on employment outcomes at 12 months	PS + ASD treatment: an intensive 9‐month Project SEARCH model during the last year of high school, consisting of three 10–12‐week internship rotations, 4 h/day	Internship work tasks varied by business site and included entry‐level skills such as stocking/organizing, merchandising, clerical/office support, basic patient care, food service, event setup, customer service, and housekeeping. Autism‐specific instructional supports included visual schedules/cues, video prompts, task analysis of multistep work routines, and ABA‐based systematic instruction/behavior supports.	CIE vocational index for adults with ASD employed participants reported job details (business, hire date, title, wage, weekly hours, main tasks, and benefits)	14 unique internship experiences were developed across seven on‐base partner organizations. At 12 months, 5/6 treatment participants achieved CIE (83.3%); four of those jobs were federal positions. 0/8 waitlist participants achieved CIE in the same period (one worked in a sheltered workshop). Treatment wages ranged $8.00–$11.00/h with 20–40 h/week, and 3/5 employed participants received benefits.
Peijen et al. 2021	To examine the impact of participation in an employer‐based work‐experience program (Philips Employment Scheme/WGP) on subsequent months in employment (any employment) and months in employment with a competitive salary, compared with a matched control group entitled to supported employment services, over up to 5 years postprogram.	1–1.5 years, typically 24–32 h/week	Not standardized or fully enumerated (training “type” not registered). Program components included temporary paid work placement within Philips, compulsory courses (career planning, professional network building, and job‐application skills), creation of a work‐conditions/workload portfolio, on‐site job coach support (including communication/self‐advocacy at work), and optional mindfulness‐based therapy; trajectories individualized.	Months in employment during the follow‐up period (employment in general, including sheltered employment where applicable). Months in employment with a “competitive salary,” operationalized as having an observed wage at or above an expected wage benchmark (estimated from Dutch labor‐market wage models used by the authors).	Participation in WGP was associated with a significant +29% increase in employment over the post‐5‐year period versus control (reported as ~13.3 additional months employed over 5 years). No significant overall effect on competitive‐salary employment across 5 years; a positive effect appeared only in the postsecond year (reported as ~3.5 months competitive‐salary employment in that year), but the difference was not sustained thereafter.
Hayward et al. 2022[30]	To examine the efficacy of Australian Disability Employment Services (DES) for autistic jobseekers, triangulating perspectives of autistic people, family members, and DES employees, and to generate suggestions to inform DES reform.	NR	Participants completed ratings + narrative responses about DES experience (autistic people/parents) or confidence/knowledge and barriers/enablers (DES staff). Questions included perceived helpfulness, understanding of workplace needs, barriers/enablers to placement and maintenance, and messages to employers.	(1) Online survey (Qualtrics) and/or 60‐min interview. (2) Standardized study questions with 7‐point Likert ratings (e.g., helpfulness, confidence/ability, understanding of needs, and autism knowledge/satisfaction) plus open‐ended explanations	Service users rated DES providers very low for ability to work with autistic clients, understanding of needs, and autism knowledge; DES staff self‐rated substantially higher, with significant discrepancies between groups. Only about one‐third of service users reported DES had helped place them into employment. Qualitative findings emphasized the need for greater flexibility in DES policy, and autism‐specific training/education for DES staff, along with improved employer engagement and a more person‐centered approach.
Kumazaki et al. 2022[31]	The purpose of this study was to examine the feasibility and effectiveness of a robot‐mediated job interview training intervention for individuals with ASD, with the aim of improving job interview performance, communication skills, and interview‐related confidence while reducing anxiety during employment preparation.	25 sessions, 5 weeks	Participants took part in mock online job interviews using virtual robots. They rotated through three roles: interviewee, interviewer, and evaluator. Each session included a first interview, a feedback discussion, and a second interview, allowing participants to practice skills and understand different perspectives. Before and after the intervention, participants completed a mock online interview with a human professional interviewer to assess change.	Subjective tools included self‐report ratings of self‐confidence, motivation, and understanding of the interviewer′s perspective using Likert scales. Objective tools included expert‐rated assessments of verbal competence, nonverbal competence, and overall interview performance based on video‐recorded mock interviews. Additional clinical measures included the Autism Spectrum Quotient—Japanese version (AQ‐J), Liebowitz Social Anxiety Scale (LSAS), ADHD Rating Scale (ADHD‐RS), and IQ tests (WAIS or WISC).	Results showed significant improvements after the intervention. Participants demonstrated higher self‐confidence, increased motivation, and better understanding of the interviewer′s perspective. Objective ratings showed improvements in verbal skills, nonverbal communication, and overall interview performance, including sincerity, enthusiasm, speaking speed, response timing, and rapport. No significant relationship was found between improvements and IQ, autism traits, social anxiety, or ADHD symptoms, suggesting benefits across participant profiles.
Moody et al. 2022[32]	Aims: To address this gap, the current study is aimed at testing the feasibility, acceptability, and efficacy of a novel college to career intervention program, PEERS for Careers.	10 weeks	Participants attended: ∗ Two 90‐min sessions per week ∗ 10 weeks total core tasks included: ∗ Didactic lessons on employment skills ∗ Role‐play demonstrations ∗ Behavioral rehearsal exercises ∗ Weekly homework assignments with a career coach Skill areas trained: ∗ Choosing a career ∗ Resume development ∗ Interviewing skills ∗ Workplace communication ∗ Navigating workplace culture ∗ Stress management and organization ∗ Disclosure and accommodations ∗ Handling conflict and bullying ∗ Networking and informational interviews.	Participants were assessed at preintervention, postintervention, and 10‐week follow‐up using: ∗ test of employment social skills (TESS)—primary outcome measure ∗ Social Responsiveness Scale‐2 (SRS‐2)—autism features ∗ ADOS‐2—diagnostic confirmation ∗ WASI‐II—cognitive ability ∗ Employment and intervention surveys (employment status and preparedness, satisfaction).	Participants demonstrated higher self‐confidence, increased motivation, and better understanding of the interviewer′s perspective.
Brighenti et al. 2023[33]	The purpose of this study was to evaluate the effectiveness of a multidisciplinary training program, combining neuropsychological and social skills training within an individual placement and support (IPS) model, in improving job‐related skills and employment outcomes for autistic individuals.	5 weeks	Participants engaged in multiple coordinated activities: 1. Career guidance and job orientation ∗ Identification of interests, skills, and vocational goals ∗ CV review and job‐search preparation 2. Internships (paid traineeships) ∗ Placement in real work environments ∗ Employer awareness and on‐site support 3. Cognitive training ∗ 18 weekly sessions (90 min each) ∗ Focus on attention, executive functions, memory, and inhibitory control ∗ Use of compensatory and restorative strategies 4. Social skills training ∗ 10 sessions (75 min each) ∗ Based on BST: instruction, modeling, practice, feedback ∗ Focus on workplace communication, interviews, and social interaction 5. Individualized support ∗ Additional tailored interventions when required.	A battery of standardized neuropsychological, psychological, and adaptive functioning measures, administered pre‐ and postintervention: ∗ RBANS—cognitive functioning ∗ Frontal assessment battery (FAB)—executive functions (especially inhibitory control) ∗ ABAS‐II—adaptive behavior ∗ SRS‐2—autism‐related social responsiveness ∗ BDI‐II—depressive symptoms ∗ STAI‐Y – anxiety ∗ Cognitive Failures Questionnaire (CFQ) ∗ Work‐skills and sensory‐profile checklist Qualitative feedback questionnaire Employment and internship status at follow‐up	Social cognition and cognitive strains may also have a significant impact on working life.
Kahng et al. 2023[34]	The purpose of this study was to evaluate the effectiveness of an individualized, remotely delivered BST program in teaching adults with ASD to successfully navigate job interviews, with a focus on improving interview performance, response quality, and generalization to novel interviewers.	The intervention duration was individualized and mastery‐based.	Participants completed repeated mock job interviews consisting of six standardized interview questions, practiced answering and asking job‐relevant questions, received instruction, modeling, rehearsal, and feedback, and progressed through increasingly intensive BST conditions until mastery criteria were met.	Mock job interviews conducted via secure video conferencing platforms (Zoom or WebEx), with responses scored using a structured 0–3 rating scale for answering interview questions and asking appropriate questions; social validity questionnaires were also administered.	All participants demonstrated improvement in job interview performance, with most reaching mastery criteria following individualized BST with feedback; pre‐ to posttraining interviews conducted by a novel career development expert showed substantial gains in interview scores, increased confidence, and improved quality of answers and questions, supporting the effectiveness of remote, individualized BST for teaching interview skills to adults with ASD.
Wehman et al. 2022[35]	To test the impact of PS + ASD delivered on a military base on CIE outcomes for military dependent/connected youth with ASD, compared with high school as usual.	It consists of a 9‐month intensive internship model delivered on the military base, with three 10–12‐week internship rotations.	Internship work duties varied by site and were documented via outcome survey (job title/duties). Employment outcomes spanned multiple industries; reported examples include hospitality/food service, retail, healthcare/social assistance, arts/entertainment/recreation, and advisory/information services, including federal positions on base.	Researcher‐made baseline survey (demographics, prior work, CBET hours, etc.). Researcher‐made outcomes survey at 12 and 18 months (employment status, job details, wage, weekly hours, benefits, support needs, etc.). SRS‐2 (Adult) SIS‐A.	PS + ASD outperformed high school as usual. At 12 months, about 61.9% of PS + ASD participants were employed (CIE), whereas 0% of the high‐school group achieved CIE; at 18 months, 60% of PS + ASD participants were employed and again 0% CIE in the high‐school group. Employed PS + ASD participants worked about 24.4 h/week at $9.38/h at 12 months; at 18 months about 25.7 h/week at $8.82/hour. Federal employment among employed PS + ASD participants increased from 46.2% (12 months) to 58.3% (18 months).
Ashburner et al. 2024[36]	To explore perceptions of an autism‐specific, client‐led employment program (Autism EmployABLE) regarding: (1) helpfulness + areas to improve and (2) clients′ happiness, confidence, and independence after gaining employment. Ashburner‐et‐al‐2024‐in‐search‐…	Not fixed‐dose (no single standardized “X weeks/Y sessions” reported). Program described as client‐led and ongoing as needed, beginning with goal setting and development of individualized “roadmaps” over several sessions, then job search/application/interview support, and job coaching that continues as needed after job start.	Client‐led goal setting (using a card‐sort goal tool), building “roadmaps” describing strengths/support needs, employer engagement/autism awareness sessions, job searching/applications, practice interviews + interview feedback, negotiation of accommodations, and ongoing postplacement support/job coaching.	Semistructured interviews.	Almost all participants viewed Autism EmployABLE as helpful and better aligned to person–job fit than generic disability employment services; perceived benefits included improved confidence, independence, and satisfaction. Outcomes for the 15 clients: 13 obtained some form of employment (10 ongoing paid; three short‐term contracts), one entered vocational training, and one did volunteer work. Suggested improvements included peer connection opportunities, industry‐specific mentoring, written materials, and aptitude testing for some roles.
Lousky et al. 2024	The study is aimed at examining whether participation in the Roim Rachok Training Course (RRTC) led to improvements in adaptive behavior and social communication skills among cognitively able autistic young adults preparing for vocational integration, particularly within military and future civilian employment contexts.	12 weeks	Participants completed a 12‐week full‐time training course, conducted 5 days per week for 7 h per day. The program included vocational training in digital, technical, or visual fields and structured employment‐preparedness activities focusing on social communication, executive functioning, adaptive living skills, emotional regulation, teamwork, and workplace behavior. Training was delivered by a multidisciplinary team including speech‐language pathologists, occupational therapists, psychotherapists, and vocational instructors.	Adaptive behavior was assessed using the Adaptive Behavior Assessment Scale–Second Edition (ABAS‐II). Autism characteristics were measured with the Social Responsiveness Scale–Second Edition (SRS‐II). Social cognition and communication were evaluated using the Faux Pas Test, the Empathy Quotient (EQ), the Friendship Quality Scale, and a structured conversation task based on the Yale in Vivo Pragmatic Protocol (YiPP). Cognitive ability was assessed at baseline using selected subtests of the WAIS‐III.	Following the intervention, participants showed significant improvements in adaptive behavior across conceptual, social, and practical domains. Social communication interaction difficulties, as measured by the SRS‐II, decreased significantly. Participants demonstrated improved social cognition, including better detection and interpretation of socially inappropriate statements and increased emotional empathy. These improvements were observed across all vocational tracks, with medium to large effect sizes, indicating that the RRTC had a broad positive impact regardless of specific vocational specialization.
Randolph et al. 2025[37]	To explore autistic adults′ self‐reported experiences after completing employment preparation/transition programs, identify unmet needs, and understand perceived impact on employment outcomes.	Not a single standardized intervention. Participants reflected on 29 employment‐preparation programs across 16 autistic adults (~60% attended > 1 program). Program “dose” varied widely by program type: University‐based programs typically met ≥ 3×/week; community job‐skills classes met 1–2×/week for several months; other community agencies met weekly to monthly and lasted from a few months to several years.	Participated in ~30‐min semistructured interviews, answering questions about programs attended, skills learned, what was/was not helpful, remaining needs, employment status, and work hours.	Semistructured interview guide (26 core questions) covering program participation, perceived helpfulness, skills learned/missed, employment status/hours.	Participants reported overall satisfaction with programs and many attributed job obtainment to program participation. Most were employed at interview (primarily part‐time). Four themes: (1) Job skills (resume/cover letters; ongoing difficulty with interviewing; desire for job‐specific/tech skills), (2) Job experience and coaching (value of exposure and coaching; concerns about overinvolvement and desire for autonomy), (3) Peer relationships (benefits of peer connection; difficulty maintaining friendships postprogram; desire for networking/social skills), and (4) Goal setting (programs helped set/reach goals but follow‐through challenges remained).

*Note:* Table 2 reproduces the extracted intervention, assessment, and outcomes fields in a structured format suitable for manuscript appendices or supporting information.

#### 4.2.1. Theme1: Integrated supported employment models and work‐based learning pathways

A dominant pattern across the literature was the use of comprehensive supported employment approaches designed to move participants into competitive integrated employment through structured pathways that combine placement, coaching, and workplace coordination. This theme was evident in programs emphasizing internship‐to‐employment pipelines, customized employment, and individual placement and support (IPS) adaptations.

##### 4.2.1.1. Internship‐Based Supported Employment as a Structured Pathway to Employment.

Several studies implemented multiphase models anchored in work‐based learning or internships followed by job placement and follow‐along support. The Project SEARCH + ASD supports line of studies consistently represented this structure [15, 26, 29, 35]. Across these studies, the intervention pathway typically combined intensive, real‐world internship experiences with systematic on‐site coaching and interagency coordination, with the explicit goal of translating internship performance into paid employment. Where quantifiable outcomes were reported in the extracted results, competitive employment outcomes were generally favorable; for example, Schall et al. (2015) reported strong employment indicators and reduced job‐development demands for participants who completed the internship‐based pathway prior to supported employment [15].

##### 4.2.1.2. Customized Employment and Individualized Job Matching.

One RCT explicitly centered on customized employment with staged discovery/assessment, job carving, and employer negotiation [16]. This approach was characterized by intensive early supports (discovery and situational assessment) followed by stabilization as job fit improved, aligning with the broader emphasis on individualized pathways.

##### 4.2.1.3. IPS‐Informed Models and Rapid Placement With Ongoing Supports.

IPS‐based supported employment adaptations for autistic participants appeared as a smaller but distinct stream [18, 33]. These studies emphasized rapid job search/placement aligned with preferences, paired with continuing support and coordination with broader services. Extracted results described positive employment attainment trajectories and employer satisfaction where measured.

Interpretive summary for Theme 1: Across these supported employment pathways, the “active ingredients” repeatedly reflected (a) structured work exposure, (b) individualized job matching, and (c) sustained support/coordination—suggesting that interventions addressing both placement and workplace maintenance were foundational in this literature.

#### 4.2.2. Theme 2: Targeted work‐readiness and skill acquisition interventions

A second major theme captured interventions that targeted specific work‐readiness skills, especially job interview performance, workplace social communication, and task execution, often delivered as time‐limited training programs or mastery‐based skill instruction.

##### 4.2.2.1. Job Interview Performance as a Primary Intervention Target.

Multiple studies focused on improving interview competence through simulated practice, behavioral skills training (BST), or technology‐mediated rehearsal [17, 25, 28, 31, 34]. Across this cluster, extracted outcomes consistently pointed to improvements in interview‐related behaviors (e.g., response quality, timing, rapport, and nonverbal communication). Technology‐mediated formats (e.g., robot‐mediated interviews and secure video‐based mock interviews) were generally reported as feasible/acceptable and were used to increase standardization and repetition.

##### 4.2.2.2. Social/Soft Skills and Broader Psychosocial Readiness.

A second substream targeted workplace‐relevant social functioning and soft skills using structured curricula and group‐based training [19–21, 23, 27, 32]. Studies in this group commonly emphasized instruction and practice in workplace conversations, peer/supervisor interactions, self‐management, and problem‐solving. Where extracted results included downstream outcomes, some studies reported employment‐related gains alongside social outcomes [27].

##### 4.2.2.3. Task‐Specific Vocational Instruction and on‐the‐Job Coaching Strategies.

A smaller set of studies focused on training discrete vocational tasks or coaching strategies using structured instruction (e.g., task analysis, prompting, and peer‐mediated coaching) [24, 29], as part of internship routines, plus other program studies incorporating systematic instruction elements. This subtheme reflected an emphasis on measurable skill acquisition and independence within specific work routines.

Interpretive summary for Theme 2: Work‐readiness interventions were highly heterogeneous but converged on a shared logic: improving “microlevel” competencies (interview behaviors, social communication, and task performance) as proximal outcomes that may enable employment access and maintenance.

#### 4.2.3. Theme 3: Implementation context, stakeholder perspectives, and sustainability considerations

A third theme reflected the consistent influence of contextual and implementation factors, including stakeholder perceptions, service quality, employer engagement, and the extent to which supports were autism‐specific and individualized. This theme was most explicit in qualitative/mixed‐methods studies [22, 30, 36, 37] and was also echoed in program evaluations describing coordination demands and workplace implementation features.

##### 4.2.3.1. Autism‐Specific Tailoring Versus Generic Employment Services.

Studies examining stakeholder experiences highlighted that autism‐specific programs were often perceived as more relevant and supportive than generic employment services [22, 36]. In contrast, mixed‐methods evidence regarding mainstream disability employment services indicated perceived gaps in autism responsiveness and a need for reform toward more person‐centered, autism‐informed practice [30].

##### 4.2.3.2. Key Facilitators: Individualized Supports, Mentoring, and Employer Engagement.

Across studies, frequently cited facilitators included individualized planning, mentoring/coaching, clear written materials, and workplace accommodations [36], also program‐based supported employment studies [15, 16, 26, 35]. Employer engagement and the quality of job coaching/support coordination emerged as recurring determinants of successful implementation.

##### 4.2.3.3. Sustainability Challenges and “What Happens After the Program”.

Qualitative accounts underscored that program participation could increase confidence and goal clarity but that follow‐through and longer‐term maintenance could be challenging without sustained supports [37]. This aligns with broader patterns in the dataset: Interventions varied substantially in duration, intensity, and follow‐up measurement, limiting conclusions about long‐term employment stability and job quality across contexts.

Interpretive summary for Theme 3: The effectiveness of employment interventions was repeatedly framed as contingent on implementation quality—particularly autism‐specific tailoring, employer engagement, and sustained support capacity—rather than on curriculum content alone.

## 5. Discussion

### 5.1. Overview of Principal Findings

This scoping review mapped 26 studies (2015–2025) addressing vocational training and employment‐related interventions for autistic adults and identified substantial diversity across intervention models, settings, dosage, and outcome measurement. From an OT perspective, the discussion below emphasizes these findings because vocational rehabilitation and work participation are established areas of OT practice, and OT contributes a distinct lens to employment support by translating intervention targets into occupational performance and participation outcomes, analyzing person–environment–occupation fit and job task demands, and designing individualized workplace strategies (e.g., environmental modifications, accommodation planning, routine/habit formation, and coaching) that enable sustainable work participation and progression [39–41].

Most studies were published from 2019 to 2025, suggesting increased research activity in the past several years. The qualitative synthesis indicated three recurring patterns in the intervention landscape: (1) integrated supported employment and work‐based learning pathways, (2) targeted work‐readiness and discrete skill acquisition interventions (notably job interview training and workplace social communication), and (3) the central role of implementation context, stakeholder perspectives, and sustainability considerations. Quantitatively, studies were concentrated in the United States and primarily occurred in workplace/community settings; intervention “dose” was inconsistently reported, and outcome measurement relied heavily on self‐report and employment indicators, with fewer studies using observed performance measures or implementation fidelity metrics. Together, these findings position the current evidence base as broad but uneven: The literature includes multiple intervention approaches with generally employment‐oriented targets, yet limited consistency in measurement and reporting constrains cross‐study comparison and practical translation to occupational performance outcomes.

### 5.2. Disciplinary Leadership and Interdisciplinary Representation

Primary investigators most frequently represented psychology/psychiatry/clinical sciences, with additional contributions from rehabilitation/vocational fields, behavior analysis, and education. Rather than indicating an absence of interdisciplinary work, this distribution descriptively indicates where the published research leadership in this topic area has most often been situated within the included sample. The diversity of investigator backgrounds is consistent with the multifactorial nature of employment participation for autistic adults, which spans individual skills, environmental demands, service systems, and policy contexts. From an OT perspective, this distribution underscores an opportunity for future research to more explicitly report disciplinary contributions when interventions target occupational performance, participation, and person–environment–occupation fit. Clearer reporting of team composition and disciplinary roles would also improve interpretability of intervention “ingredients” (e.g., task analysis, environmental modification, and coaching practices) and support replication across service contexts.

### 5.3. Intervention Landscape: Supported Employment and Work‐Based Learning Pathways

Theme

One highlighted that a dominant stream of studies used integrated supported employment models designed to move participants toward competitive integrated employment through structured sequences of work exposure, job matching, and ongoing supports. This pattern aligns with earlier syntheses emphasizing the role of on‐the‐job supports in vocational outcomes for autistic adolescents and adults. For example, Taylor et al.′s review of vocational interventions for youth and young adults with ASD (13–30 years) identified few studies and noted that available interventions largely focused on on‐the‐job supports and supported employment, whereas the evidence base was limited and of low quality at that time [42].

In the current review, supported employment approaches appeared in more contemporary and structured forms, including internship‐to‐employment pipelines (e.g., Project SEARCH + ASD supports), customized employment with individualized job carving, and IPS‐informed adaptations. This pattern is also consistent with RCT‐focused evidence syntheses indicating that, among the relatively small number of autism‐including RCTs, Project SEARCH + ASD supports shows evidence of benefit for open/competitive employment outcomes [43]. Importantly, as a scoping review, the present work does not estimate pooled effects; however, supported employment and work‐based learning pathways were reported in 11 of 26 studies (42.3%), spanning multiple designs (e.g., RCTs, pilot, quasi‐experimental, and qualitative studies) and delivery contexts (community, workplace, and military‐base settings), suggesting that “work‐based learning plus support” is a recurring organizing logic in the field.

For OT, these models closely map to core practice emphases grounded in established occupation‐based frameworks: enabling work participation by analyzing person–environment–occupation fit, job/task demands, and environmental supports/accommodations, while also addressing role expectations and routines and aligning employment goals with the individual′s values, preferences, and strengths [40, 44–48]. The current evidence map suggests that future studies could strengthen reporting on how job matching decisions are made, what workplace accommodations are implemented, and which components are delivered by which professionals (e.g., coaching, environmental modification, and self‐management supports). Such specification would enhance replicability and facilitate translation into OT service pathways across vocational rehabilitation, community employment services, and integrated care models.

### 5.4. Targeted Work‐Readiness and Skill Acquisition Interventions, Including Technology‐Mediated Delivery

Theme

Two captured a second cluster of interventions focused on discrete, trainable work‐readiness skills. Job interview performance was a particularly common target, addressed through BST, structured rehearsal, and technology‐mediated platforms (e.g., robot‐mediated mock interviews, remote instruction, and virtual interview training tools). This thematic pattern aligns with earlier vocational intervention reviews that categorized adult ASD employment interventions as BST, video‐based instruction, and self‐management procedures, while noting underreporting of generalization and social validity [49]. More recent studies extend these approaches into technology‐assisted delivery formats and into outcomes that are closer to employment access (e.g., interview performance metrics, employer ratings, and job access indicators), but the literature remains heterogeneous in both intervention dose and outcome selection.

Technology‐mediated interview training provides a clear example of how the field is evolving. Controlled and feasibility trials have evaluated virtual interview training and VR‐based job interview training approaches in autistic transition‐age groups, reporting improvements in interview skill outcomes and other proximal indicators (e.g., self‐efficacy and interview‐related anxiety) as a measured outcome in some studies [6, 50]. A recent realist review has also examined how VR interventions might operate within recruitment and early employment contexts, emphasizing the importance of context and mechanism specification [51]. For the present scoping review, the key contribution is not to claim superiority of technology‐based methods, but to document that technology is increasingly used to standardize practice opportunities, increase repetition, and support implementation in settings where in‐person rehearsal may be resource‐intensive.

From an OT standpoint, these interventions can be interpreted as targeting “microlevel” performance components that contribute to broader occupational outcomes (employment participation and role performance). Future research would benefit from consistently linking these proximal targets to occupationally meaningful endpoints that reflect contemporary work trajectories—not only job attainment and retention, but also sustainable participation and career development (e.g., work role competence, job satisfaction and wellbeing, workplace fit and accommodation success, participation patterns over time, and career advancement or positive job transitions to better‐fit or higher‐level roles). Studies should also report whether gains generalize beyond training contexts to real interviews and everyday job routines, and whether outcomes are maintained during workplace changes and progression over time. This linkage is particularly important because OT practice prioritizes functional performance and participation, yet the current literature varies widely in whether those endpoints are mesured directly. This variability may partly reflect disciplinary differences in how outcomes are conceptualized and measured, given that many studies were not led by occupational therapists.

### 5.5. Implementation Context, Stakeholder Perspectives, and Sustainability

Theme

Three extends prior syntheses by showing that contextual and implementation considerations are not incidental but recurrently emphasized in the contemporary literature, particularly in qualitative and mixed‐methods studies. This emphasis is practically salient because intervention feasibility and sustainability are shaped not only by program content but also by the surrounding service ecology—specifically, how each country funds disability employment supports, the degree to which formal systems (e.g., vocational rehabilitation and supported employment infrastructure) are established, and the incentives and responsibilities placed on employers and service providers. In this review, many studies were conducted in the United States, and the prominence of certain models and outcomes should therefore be interpreted with attention to the US policy and funding context, which may enable particular pathways (and constraints) that do not translate directly to other settings. Earlier reviews of vocational interventions for ASD highlighted that the evidence base was small and often limited by weak study quality and incomplete reporting (including limited fidelity measurement), even when on‐the‐job supports were central [42]. Skills training–focused syntheses have catalogued intervention procedures and outcomes across many experiments, commonly identifying video‐based and behavioral instructional components, but they necessarily place less emphasis on how broader service systems, employers, and ongoing supports shape real‐world feasibility and sustainability [52]. The present scoping review complements those contributions by mapping how recent studies increasingly document stakeholder perspectives, autism‐specific tailoring, and “what happens after the program” considerations as recurring elements of the intervention landscape, while underscoring the need for more geographically diverse research that explicitly describes funding mechanisms, service delivery structures, and implementation conditions to support meaningful cross‐country interpretation.

### 5.6. Outcome Measurement Heterogeneity and Implications for Occupational Performance Assessment

Across studies, outcome measurement most frequently relied on self‐report tools and employment/vocational outcomes, with nearly half also using qualitative methods. Fewer studies reported observed performance measures, and only a small subset reported fidelity/implementation outcomes. This distribution has two implications. First, heavy reliance on self‐report measures can be appropriate for constructs such as perceived work readiness, self‐efficacy, and satisfaction, but limits comparability when different tools are used across studies and when objective or observed performance is not concurrently measured. Second, limited fidelity measurement constrains interpretation of what was actually delivered, by whom, and with what level of adherence—particularly relevant for complex supported employment models and multicomponent programs.

Prior reviews have similarly highlighted measurement and reporting challenges. For example, Anderson et al. emphasized limited reporting of social validity and generalization in adult vocational intervention studies, and Weld‐Blundell et al. noted gaps in study quality and consistent measurement across vocational intervention RCTs, particularly for autism‐including studies [43, 49]. Campanaro et al.′s review of vocational training interventions also summarized generally positive outcomes across studies while reflecting the broader need for clearer specification of outcomes and methods in this literature [52].

For OT research, these findings underscore the value of moving toward clearer outcome frameworks that distinguish (a) participation outcomes (job attainment, hours worked, and retention), (b) occupational performance outcomes (work task performance, role competence, and productivity/quality metrics where feasible), (c) contextual outcomes (accommodation processes, workplace supports, and employer outcomes), and (d) implementation outcomes (fidelity, acceptability, feasibility, and cost). Consistent reporting across these domains would allow future reviews to compare interventions on outcomes that align with OT′s focus on participation and performance in real contexts.

### 5.7. Geographic Concentration and Implications for Transferability

Of the 26 included studies, 14 (53.8%) were conducted in the United States, with smaller numbers from Japan (*n* = 4) and Australia (*n* = 3). This concentration may partly reflect the US context, where disability employment is supported by comparatively well‐established legal and policy frameworks (e.g., the Americans with Disabilities Act′s employment protections and reasonable accommodation requirements, and the Workforce Innovation and Opportunity Act′s emphasis on workforce participation and vocational rehabilitation services), which can facilitate implementation, resourcing, and evaluation of vocational programs. It may also be influenced by the US origin and diffusion of specific service models frequently studied in this field (e.g., Project SEARCH). Project SEARCH is an employment preparation model for individuals with disabilities that originated at Cincinnati Childrenâ€™s Hospital Medical Center and has since expanded to multiple program sites internationally. However, if eligibility was limited to English‐language publications, the apparent geographic imbalance should also be interpreted as a limitation because relevant evidence from other countries may be underrepresented due to local‐language dissemination and practice reporting, introducing potential language/publication bias [53–57].

### 5.8. How This Review Extends Prior Syntheses

Compared with earlier reviews that characterized the evidence base as small, heterogeneous, and often methodologically weak [39], the present scoping review maps a larger body of contemporary work (26 studies, 2015–2025), including a notable proportion of RCTs alongside single‐case experimental, qualitative, and mixed‐methods designs. This growth aligns with the broader trajectory of increasing research activity in recent years while also confirming that heterogeneity in intervention models and outcome measurement remains a defining feature of the field. Differences across reviews are also partly attributable to scope and eligibility criteria: For example, Taylor et al. identified only six papers evaluating supported employment/vocational interventions for adolescents and young adults with ASD and rated overall study quality as poor [42], whereas the Campbell update by Fong et al. restricted inclusion to randomized/quasi‐experimental studies reporting employment outcomes and identified only three eligible studies (including Project SEARCH + ASD supports and VR job interview training) [58]. By design, our broader scoping approach does not adjudicate effectiveness but instead clarifies how the literature is currently organized into recurring pathways and where evaluation emphasis has concentrated.

Relative to reviews focused on discrete behavioral vocational training strategies, this review integrates three strands into a coherent thematic map: (1) supported employment and work‐based learning pathways, (2) targeted work‐readiness and discrete skill acquisition interventions (including technology‐assisted delivery), and (3) implementation context, stakeholder perspectives, and sustainability considerations [49, 52]. Consistent with skills‐training syntheses, commonly reported components such as video modeling, BST, feedback, and prompting appear prominently within the work‐readiness/skill acquisition literature [52], and video‐based reviews similarly emphasize job performance targets more often than job search skills [59]. At the same time, our synthesis highlights a persistent measurement challenge across intervention types: Outcomes frequently rely on self‐report and employment indicators, with fewer studies using observed performance measures or implementation fidelity metrics, limiting cross‐study comparison and practical translation. Finally, extending earlier calls for better intervention description and fidelity reporting [42], the present review shows that recent qualitative and mixed‐methods studies increasingly foreground autism‐specific tailoring, employer engagement, and postprogram support as recurring features to document alongside outcomes, underscoring implementation quality and sustainability as core considerations in the contemporary evidence base [42, 52].

### 5.9. Limitations

Several limitations should be considered when interpreting these findings. First, as a scoping review, this work is aimed at mapping the breadth and characteristics of the literature rather than to estimate effects or determine comparative effectiveness; conclusions should therefore remain descriptive. Second, heterogeneity in intervention reporting, dose specification, and outcome measurement limited the degree to which interventions could be compared on common metrics. Third, many studies had small sample sizes and were conducted in specific service contexts, which may restrict generalizability. Finally, although the thematic synthesis identified recurring patterns, the relative frequency of themes does not necessarily indicate effectiveness, and some intervention components may be underrepresented due to reporting practices.

### 5.10. Implications for OT Research and Practice

The mapped evidence indicates that vocational interventions for autistic adults frequently target employment participation through supported employment pathways, discrete work‐readiness skill training, and context‐sensitive implementation supports. For OT, the practical implication is that published interventions commonly align with OT′s core emphasis on enabling occupational performance and participation in work roles, yet the literature would benefit from more consistently linking intervention targets to occupationally meaningful outcomes (e.g., observed work performance, participation, and person–environment–occupation fit) and from clearer reporting of intervention components and implementation processes to support replication and translation to practice (e.g., TIDieR) [40, 44, 45, 60]. Future studies that incorporate consistent measurement frameworks, specify intervention ingredients and professional roles, and evaluate longer‐term employment stability and job quality would strengthen the field′s ability to translate evidence into scalable services across contexts.

## 6. Conclusions

This scoping review mapped 26 studies published between 2015 and 2025 on vocational training and employment‐related interventions for autistic adults and characterized a rapidly growing but heterogeneous evidence base. Across the included literature, three recurring patterns were identified: integrated supported employment and work‐based learning pathways; targeted work‐readiness and discrete skill acquisition interventions (particularly interview and workplace communication skills); and the central influence of implementation context, stakeholder perspectives, and sustainability. Quantitative mapping further indicated that studies were concentrated in a small number of countries (most commonly the United States), were frequently delivered in workplace/community contexts, and varied widely in intervention duration and intensity, with inconsistent reporting of “dose” across studies.

From an OT perspective, the current evidence map indicates that interventions commonly aim to improve participation in the worker role, but the field remains constrained by heterogeneity in outcome measurement and limited use of observed vocational performance and implementation fidelity metrics. As a descriptive synthesis, this review does not determine comparative effectiveness; rather, it clarifies how the literature is currently organized and where evaluation emphasis has clustered. Future research would be strengthened by more consistent specification of intervention components and delivery roles, standardized reporting of intervention exposure, and outcome frameworks that more directly capture occupational performance and participation alongside implementation and sustainability outcomes.

## Author Contributions

H.A.H. contributed to conceptualization, methodology, literature search, screening, data extraction, qualitative synthesis, and drafting the manuscript. H.A.H. and D.F. independently screened records and extracted data. R.M. served as the corresponding author and overall supervisor for the study, providing methodological oversight, resolving disagreements during screening and extraction, guiding interpretation and synthesis validation, and overseeing manuscript development and final approval.

## Funding

This study was supported by the Tokyo Metropolitan University (10.13039/501100010236).

## Disclosure

All authors reviewed and approved the final version.

## Conflicts of Interest

The authors declare no conflict of interest.

## Data Availability

The data that support the findings of this study are available on request from the corresponding author. The data are not publicly available due to privacy or ethical restrictions.

## Appendix A: Full electronic search strategies:

Database searches were conducted in two stages (June 2024 initial scoping search; November 2025 updated and expanded search). The complete database‐specific search strategies reported below correspond to the updated search executed on November 14–15, 2025, including controlled vocabulary where available.

PubMed (MEDLINE).

Run date: November 14, 2025.

**Table A1:** Full electronic search strategy for PubMed (MEDLINE), including search terms, combinations, limits, and results of the updated search conducted on 14 November 2025.

Search line	Search strategy (as executed)	Run information
Search #1	“autism spectrum disorder”[MeSH Terms] OR “autistic disorder”[MeSH Terms] OR “autis∗”[Title/Abstract] OR “asperger∗”[Title/Abstract] OR “pervasive developmental disorder∗”[Title/Abstract]	Results: 86,490; Time: 04:41:12 (JST); Date: 2025/11/14
Search #2	“rehabilitation, vocational”[MeSH Terms] OR “employment”[MeSH Terms] OR “employment, supported”[MeSH Terms] OR “return to work”[MeSH Terms] OR “workplace”[MeSH Terms] OR “supported employ∗”[Title/Abstract] OR “individual placement support”[Title/Abstract] OR “IPS”[Title/Abstract] OR “job coach∗”[Title/Abstract] OR “job coaching”[Title/Abstract] OR “job placement”[Title/Abstract] OR “vocational training”[Title/Abstract] OR “prevocational”[Title/Abstract] OR “work readiness”[Title/Abstract] OR “work integration”[Title/Abstract] OR “work based learning”[Title/Abstract] OR “on the job training”[Title/Abstract] OR “school‐to‐work”[Title/Abstract] OR “transition to work”[Title/Abstract] OR “competitive integrated employment”[Title/Abstract] OR “customized employ∗”[Title/Abstract]	Results: 132,360; Time: 04:49:59 (JST); Date: 2025/11/14
Search #3	(“autism spectrum disorder”[MeSH Terms] OR “autistic disorder”[MeSH Terms] OR “autis∗”[Title/Abstract] OR “asperger∗”[Title/Abstract] OR “pervasive developmental disorder∗”[Title/Abstract]) AND (“rehabilitation, vocational”[MeSH Terms] OR “employment”[MeSH Terms] OR “employment, supported”[MeSH Terms] OR “return to work”[MeSH Terms] OR “workplace”[MeSH Terms] OR “supported employ∗”[Title/Abstract] OR “individual placement support”[Title/Abstract] OR “IPS”[Title/Abstract] OR “job coach∗”[Title/Abstract] OR “job coaching”[Title/Abstract] OR “job placement”[Title/Abstract] OR “vocational training”[Title/Abstract] OR “prevocational”[Title/Abstract] OR “work readiness”[Title/Abstract] OR “work integration”[Title/Abstract] OR “work based learning”[Title/Abstract] OR “on the job training”[Title/Abstract] OR “school‐to‐work”[Title/Abstract] OR “transition to work”[Title/Abstract] OR “competitive integrated employment”[Title/Abstract] OR “customized employ∗”[Title/Abstract])	Results: 530; Time: 04:50:11 (JST); Date: 2025/11/14
Search #4	((“autism spectrum disorder”[MeSH Terms] OR “autistic disorder”[MeSH Terms] OR “autis∗”[Title/Abstract] OR “asperger∗”[Title/Abstract] OR “pervasive developmental disorder∗”[Title/Abstract]) AND (“rehabilitation, vocational”[MeSH Terms] OR “employment”[MeSH Terms] OR “employment, supported”[MeSH Terms] OR “return to work”[MeSH Terms] OR “workplace”[MeSH Terms] OR “supported employ∗”[Title/Abstract] OR “individual placement support”[Title/Abstract] OR “IPS”[Title/Abstract] OR “job coach∗”[Title/Abstract] OR “job coaching”[Title/Abstract] OR “job placement”[Title/Abstract] OR “vocational training”[Title/Abstract] OR “prevocational”[Title/Abstract] OR “work readiness”[Title/Abstract] OR “work integration”[Title/Abstract] OR “work based learning”[Title/Abstract] OR “on the job training”[Title/Abstract] OR “school‐to‐work”[Title/Abstract] OR “transition to work”[Title/Abstract] OR “competitive integrated employment”[Title/Abstract] OR “customized employ∗”[Title/Abstract])) AND (y_10[Filter])	Filters: in the last 10 years; Results: 344; Time: 04:50:16 (JST); Date: 2025/11/14

MEDLINE via EBSCOhost.

Run date: November 14, 2025. Expander: Apply equivalent subjects.

**Table A2:** Full electronic search strategy for MEDLINE via EBSCOhost, including search terms, combinations, limits, and results of the updated search conducted on 14 November 2025.

Search line	Search strategy (as executed)	Run information
S1	MM (Autism Spectrum Disorder) OR MH (Autistic Disorder) OR TI (autis∗ OR asperger∗ OR “pervasive developmental disorder∗) OR AB (autis∗ OR asperger∗ OR “pervasive developmental disorder∗)	Results: 84370; Run: 2025‐11‐14 T09:53:33.551Z
S2	MH (Rehabilitation, Vocational) OR MH (Employment) OR MH (Employment, Supported) OR MH (Return to Work) OR MH (Workplace)	Results: 96606; Run: 2025‐11‐14 T09:55:29.759Z
S3	XB (supported employ∗) OR XB (individual placement and support or ips) OR XB (job placement) OR XB (vocational training OR prevocational) OR XB (work readiness) OR XB (work‐based learning) OR XB (on‐the‐job training) OR XB (school‐to‐work) OR XB (transition to work) OR XB (competitive integrated employment) OR XB (customized employ∗)	Results: 20602; Run: 2025‐11‐14 T09:58:46.779Z
S4	(MH (Rehabilitation, Vocational) OR MH (Employment) OR MH (Employment, Supported) OR MH (Return to Work) OR MH (Workplace)) OR (XB (supported employ∗) OR XB (individual placement and support or ips) OR XB (job placement) OR XB (vocational training OR prevocational) OR XB (work readiness) OR XB (work‐based learning) OR XB (on‐the‐job training) OR XB (school‐to‐work) OR XB (transition to work) OR XB (competitive integrated employment) OR XB (customized employ∗))	Results: 114975; Run: 2025‐11‐14 T09:59:16.790Z
S5	(MM (Autism Spectrum Disorder) OR MH (Autistic Disorder) OR TI (autis∗ OR asperger∗ OR “pervasive developmental disorder∗) OR AB (autis∗ OR asperger∗ OR “pervasive developmental disorder∗)) AND ((MH (Rehabilitation, Vocational) OR MH (Employment) OR MH (Employment, Supported) OR MH (Return to Work) OR MH (Workplace)) OR (XB (supported employ∗) OR XB (individual placement and support or ips) OR XB (job placement) OR XB (vocational training OR prevocational) OR XB (work readiness) OR XB (work‐based learning) OR XB (on‐the‐job training) OR XB (school‐to‐work) OR XB (transition to work) OR XB (competitive integrated employment) OR XB (customized employ∗)))	Results: 524; Run: 2025‐11‐14 T09:59:44.634Z
S6	(MM (Autism Spectrum Disorder) OR MH (Autistic Disorder) OR TI (autis∗ OR asperger∗ OR “pervasive developmental disorder∗) OR AB (autis∗ OR asperger∗ OR “pervasive developmental disorder∗)) AND ((MH (Rehabilitation, Vocational) OR MH (Employment) OR MH (Employment, Supported) OR MH (Return to Work) OR MH (Workplace)) OR (XB (supported employ∗) OR XB (individual placement and support or ips) OR XB (job placement) OR XB (vocational training OR prevocational) OR XB (work readiness) OR XB (work‐based learning) OR XB (on‐the‐job training) OR XB (school‐to‐work) OR XB (transition to work) OR XB (competitive integrated employment) OR XB (customized employ∗)))	Results: 340; Run: 2025‐11‐14 T10:03:47.657Z; Limit: 2015‐11‐14 to 2025‐11‐14

Web of Science Core Collection.

Run date: November 15, 2025. *Note:* exported search history may truncate long query strings with ellipses; the syntax is reproduced as exported.

**Table A3:** Full electronic search strategy for Web of Science Core Collection, including search terms, combinations, limits, and results of the updated search conducted on 15 November 2025.

Search line	Search strategy (as executed)	Run information
Line 1	Autis∗ (Title) OR autism spectrum disorder (Title) OR asperger∗ (Title) OR autis∗ (Abstract) OR autism spectrum disorder (Abstract) OR asperger∗ (Abstract)	Results: 106582; Run: Sat Nov 15 2025 09:29:33 GMT+0900 (Japan Standard Time)
Line 2	Supported employ∗ (Title) OR individual placement and support (Title) OR job coach∗ (Title) OR job placement (Title) OR vocational training (Title) OR work readiness (Title) OR work integration (Title) OR on‐the‐job training (Title) OR school‐to‐work (Title) OR transition to work (Title) OR competitive integrated employment (Title) OR customized employ∗ (Title) OR prevocational (Title)	Results: 10568; Run: Sat Nov 15 2025 09:33:19 GMT+0900 (Japan Standard Time)
Line 3	“supported” NEAR/2 employ∗ (Abstract) OR individual placement and support (Abstract) OR IPS (Abstract) OR “job” NEAR/2 coach∗ (Abstract) OR “job” NEAR/2 placement (Abstract) OR vocational NEAR/2 training (Abstract) OR prevocational (Abstract) OR “work” NEAR/2 readiness (Abstract) OR “work” NEAR/2 integration (Abstract) OR “on” NEAR/1 “the” NEAR/1 job NEAR/2 training (Abstract) OR school‐to‐work (Abstract) OR transition to work (Abstract) OR “competitive” NEAR/2 integrated NEAR/2 employ∗ (Abstract) OR “customized” NEAR/2 employ∗ (Abstract)	Results: 213283; Run: Sat Nov 15 2025 09:36:55 GMT+0900 (Japan Standard Time)
Line 4	#2 OR #3	Results: 219675; Run: Sat Nov 15 2025 09:37:14 GMT+0900 (Japan Standard Time)
Line 5	#1 AND #4	Results: 568; Run: Sat Nov 15 2025 09:37:42 GMT+0900 (Japan Standard Time)
Line 6	#1 AND #4 and 2025 or 2024 or 2022 or 2023 or 2021 or 2020 or 2016 or 2017 or 2018 or 2019 (Publication Years)	Results: 433; Run: Sat Nov 15 2025 09:38:04 GMT+0900 (Japan Standard Time)
